# To Adapt or Not to Adapt: The Association between Implementation Fidelity and the Effectiveness of Diabetes Self-Management Education

**DOI:** 10.3390/ijerph18084095

**Published:** 2021-04-13

**Authors:** Louise Schinckus, Stephan Van den Broucke, Gerard van der Zanden, Diane Levin-Zamir, Gabriele Mueller, Henna Riemenschneider, Victoria Hayter, Lucy Yardley, Dean Schillinger, Gerardine Doyle, Kristin Ganahl, Jürgen Pelikan, Peter Chang

**Affiliations:** 1 Centre Hospitalier Universitaire Brugmann, 1020 Brussels, Belgium; schinckuslouise@gmail.com; 2 Psychological Sciences Research Institute, Université Catholique de Louvain, 1348 Louvain-la-Neuve, Belgium; gvanderzanden@outlook.com; 3 School of Public Health, University of Haifa and Clalit Health Services, Tel Aviv 62098, Israel; diamos@inter.net.il; 4 Faculty of Medicine Carl Gustav Carus, University Hospital Dresden, Technische Universität Dresden, 01307 Dresden, Germany; gabriele.mueller@tu-dresden.de (G.M.); henna.riemenschneider@uniklinikum-dresden.de (H.R.); 5 Centre for Clinical and Community Applications of Health Psychology, University of Southampton, High Field, Southampton SO17 1BJ, UK; V.J.Hayter@soton.ac.uk (V.H.); L.Yardley@soton.ac.uk (L.Y.); 6 Department of Medicine, University of California at San Francisco, UCSF Box 1364, San Francisco, CA 94110, USA; dean.schillinger@ucsf.edu; 7 College of Business and Geary Institute for Public Policy, University College Dublin, D04 V1W8 Dublin, Ireland; gerardine.doyle@ucd.ie; 8 AKS Gesundheit GmbH, 6900 Bregenz, Austria; kristin.ganahl@aks.or.at; 9 Gesundheit Österreich GmbH (GÖG), 1010 Wien, Austria; juergen.pelikan@goeg.at; 10 Department of Occupational Medicine, Chang Bing Show Chwan Memorial Hospital, Lukang, Changhua 505, Taiwan and Department of Medicine, Chung Shan Medical University, Taichung City 40201, Taiwan; peter.chang3@gmail.com

**Keywords:** diabetes, self-management education, implementation fidelity, adherence, adaptation, intervention effectiveness

## Abstract

Self-management education (SME) is a key determinant of diabetes treatment outcomes. While SME programs are often adapted for implementation, the impact of adaptations on diabetes SME effectiveness is not well documented. This study evaluated the impact of the implementation fidelity of diabetes SME programs on program effectiveness, exploring which factors influence implementation fidelity. Data from 33 type 2 diabetes SME program providers and 166 patients were collected in 8 countries (Austria, Belgium, Germany, Ireland, UK, Israel, Taiwan and USA). Program providers completed a questionnaire assessing their adherence to the program protocol and factors that influenced the implementation. Patients answered a pre–post questionnaire assessing their diabetes-related health literacy, self-care behavior, general health and well-being. Associations between implementation fidelity and outcomes were estimated through logistic regressions and repeated measures MANOVA, controlling for potential confounders. Adaptations of the program protocol regarding content, duration, frequency and/or coverage were reported by 39% of the providers and were associated with better, not worse, outcomes than strict adherence. None of the factors related to the participants, facilitating strategies, provider or context systematically influenced the implementation fidelity. Future research should focus on individual and contextual factors that may influence decisions to adapt SME programs for diabetes.

## 1. Introduction

Diabetes is a chronic disease that requires daily decision making and self-care by the patients. Due to this need to self-manage the disease, a patient’s capacity to manage his or her disease is considered a key determinant of treatment outcomes and related costs [1]. To help people with diabetes navigate these decisions and care activities, diabetes self-management education (DSME) is generally recommended. DSME is broadly defined as the process of facilitating the knowledge, skill and ability that are required for diabetes self-care. While a wide range of DSME programs are available [2], many of which have been shown to improve health outcomes, the effectiveness of DSME depends on a variety of factors, related to the patient (motivation, level of distress, health literacy, etc.), the characteristics of the education program (e.g., content, format) and the organizational context (embeddedness of the program in a larger organization, composition and competences of the team, etc.). An additional factor that is sometimes mentioned as a determinant of DSME effectiveness is the way the program is implemented. Yet, implementation remains a peripheral issue in the literature on DSME, and there is a clear lack of research about the implementation fidelity of existing programs.

Implementation fidelity, or intervention integrity, can be defined as “the extent to which an intervention is delivered as intended” [3] and thus involves a comparison between the intervention as implemented and the original program [4]. Although the concept was already introduced in the 1970s [5,6,7], it has only recently gained traction as a research topic within the health domain [8,9,10,11]. The idea behind it is that a careful mapping of the way in which an intervention diverges from the original gives a better understanding of what works or does not work during program delivery [12]. Specifically, this helps to avoid the erroneous attribution of the absence of significant effects of an intervention to the ineffectiveness of the intervention itself, when it may in fact result from a poor implementation—a phenomenon that has been referred to as the ‘‘type III error’’ [13]. Alternatively, yet much less often considered, it may also reveal that positive outcomes are due to adaptations of an intervention. Information about the fidelity of a program’s implementation can thus help to understand why an intervention succeeded or was less effective. Evaluating implementation fidelity also makes explicit which specific components of the intervention were adapted, and how these modifications affected the outcomes of the intervention, which can help to enhance the future feasibility of implementing the intervention in a formative approach.

To operationalize implementation fidelity, three different methods have been proposed: direct observation (either participating or non-participating); indirect observation (audio or video recording); and self-reports (questionnaire or interview) by the participants and/or providers. Each of these methods has its advantages and disadvantages [14]. Observation tends to provide a more objective and accurate assessment of the program implementation but is cumbersome and costly, as it requires the observers to be trained and spend long periods of time in the field. Observation by an outsider can also influence implementation fidelity, due to the practitioners’ reactivity to observation. Self-report measures, on the other hand, are less expensive and less time-consuming but are more prone to bias due to social desirability on the part of the providers, whereas participants may be influenced by their feelings toward the provider.

Epistemologically, a distinction can be made between a critical component and a dimensional approach to measure implementation fidelity [15]. According to the former, a program consists of several core components that are essential to achieve effectiveness; therefore, assessing implementation fidelity involves checking if each of these components has been correctly delivered to the participants. The dimensional approach, on the other hand, posits that implementation fidelity is a multidimensional concept, and that each dimension can be assessed separately. There is no general consensus as to which dimensions are the most crucial for implementation fidelity, but the three that are most often considered are adherence, exposure and quality. While the critical component approach makes it possible to assess very specific aspects of the intervention, the dimensional approach allows researchers to compare the implementation fidelity of different kinds of interventions.

A combination of both approaches was proposed by Carroll et al. [16], whose conceptual model of implementation fidelity uses a dimensional approach but integrates critical components in one of the dimensions. Specifically, this model defines implementation fidelity as the provider’s adherence to the original program content (were all the core components delivered to the participants?), duration and frequency (was the intervention delivered with the frequency and duration required by the developers?) and coverage (have all the persons who should have participated in the intervention done so?). It also acknowledges that implementation fidelity—or the provider’s adherence to the original program—can be influenced by different contextual and individual factors. These include: the intervention’s complexity (i.e., its nature and comprehensiveness); the presence of facilitating strategies (e.g., a manual, training and feedback to support and standardize the implementation); the quality of delivery (i.e., the skills, attitudes and dedication of the individuals who are responsible for delivering the intervention); and the participant responsiveness (i.e., higher implementation fidelity is achieved when the participants are more enthusiastic about the intervention). Two additional influencing factors were later added by Hasson [17]: recruitment issues (i.e., the procedure for selecting and recruiting participants, reasons for non-participation and the presence or absence of specific participant subgroups); and the context or the culture and organizational structure in which the intervention takes place (e.g., positive working climate, norms to change, shared decisions, communication). The integration of the critical and dimensional approaches, as well as the consideration of different potential influencing factors, results in a comprehensive framework that can guide the assessment of implementation fidelity.

While the above-mentioned model assumes that maximum adherence to the original protocol is the best guarantee to achieve the best outcomes, this assumption is increasingly being challenged [18,19,20]. Not only is 100% fidelity rarely reached in practice, but adjustments to a program can also have a positive impact on effectiveness [21]. While maximum adherence to the initial protocol may indeed ensure that the main components of the intervention are actually delivered, certain adaptations to the intervention take the participants’ specific needs better into account and thus increase the contextual and cultural relevance of the intervention [22]. Adaptations may also address providers’ needs [19], and some interventions are even designed intentionally to allow certain adaptations [23]. From that perspective, assessing the adaptations that providers make to a program helps to map what parts of the intervention can be adapted and which ones should not be changed, in order to achieve the highest level of effectiveness.

A literature review [24] showed that implementation fidelity of diabetes self-management education programs remains largely under-investigated. Despite the importance of implementation fidelity for the practice of diabetes education, very few studies document the providers’ adherence to the original program protocol, and even fewer studies have considered the impact of provider adherence to the protocol on the effectiveness of diabetes education. Drawing on the conceptual model of implementation fidelity proposed by Carroll et al. [16], the present study aimed to assess (i) the association between implementation fidelity and the effectiveness of DSME programs, examining whether adaptations have an impact on the program outcomes; and (ii) which factors related to the participants, the provider, the delivery strategies or the context are associated with implementation fidelity. It was expected that providers would better adhere to the program protocol when (i) participants were motivated and engaged in the program; (ii) providers had sufficient knowledge about and a positive attitude towards the program’s content; (iii) manuals and feedback were available to help the providers implement the intervention; and (iv) the context for the intervention delivery was flexible and adequate.

## 2. Materials and Methods

### 2.1. Recruitment and Data Collection Procedure

This study was part of a larger international study on factors that influence the effectiveness of diabetes self-management education [25]. To assess the impact of implementation fidelity on the effectiveness of DSME programs, a pre–post comparative study design was used. DSME programs were selected from a compendium of existing programs in Austria, Belgium, Germany, Ireland, the UK, Israel, Taiwan and the USA [2]. To be selected for inclusion, programs had to: (1) target diagnosed type 2 diabetes patients; (2) be set up for the general (patient) population rather than for a specific age cohort, specific needs or a specific gender group; (3) be eligible for newly diagnosed patients as well as for patients with existing diabetes; (4) be stand-alone rather than an add-on to another program or part of a wider curriculum with (multiple) parallel programs; (5) admit new patients during the time of the baseline data collection.

For each program, patients who joined between October 2014 and June 2015 were systematically asked to participate in the study. Program staff were asked to distribute questionnaires to the patients. Patients who agreed to participate and completed an informed consent form received the pre-assessment questionnaire in a stamped envelope. Three months later, they were contacted by phone for the post-intervention questionnaire. This procedure was followed by all participating countries, except for Israel, where the baseline data were also collected through a telephone interview, and Germany, where the baseline data and the post-intervention questionnaire were collected via an e-mail survey (see Suppl. Table S1 for the number of programs, providers and participants per country).

At the end of the program, the program providers were invited to participate in a structured interview (face-to-face or by phone) to assess the implementation fidelity. Provider and patient data were linked to each other by means of a unique ID that enabled linking the intervention outcomes (patient assessment level) to the implementer’s adherence to the program (provider assessment level).

### 2.2. Participants

A total of 166 diabetes patients who participated in 16 different DSME programs completed the pre- and post-intervention questionnaires. Their responses were linked to the structured interview data of 33 providers. Among the 16 programs, three were delivered individually; the others were delivered to groups of participants. The patient sample was composed of 85 men (51.2%) and 81 women (48.8%). The mean age was 61.34 (SD = 11.562). On average, patients had 11 years of schooling (SD = 4.66), and the vast majority (150 or 90.4%) had the citizenship of the associated participating country. On a scale from 1 to 10, participants positioned their social status as average (score of 5.59, SD = 2.045).

### 2.3. Measures

To investigate the intervention outcomes, an integrated questionnaire [26] was used which measured the following aspects (see Table 1):

(a) Self-care behaviors were measured by means of the Summary of Diabetes Self-Care Activities Measure (SDSCA) [27]; this eight-point scale (0–7) assesses diet (e.g., “How many of the last SEVEN DAYS have you followed a healthful eating plan?”), exercise (e.g., “On how many of the last SEVEN DAYS did you participate in at least 30 min of physical activity?”), medication (e.g., “On how many of the last SEVEN DAYS did you take your recommended insulin injections/number of diabetes pills?”) and foot care (e.g., “On how many of the last SEVEN DAYS did you check your feet?”).
(b) *Diabetes-specific health literacy* was measured with the Diabetes Health Literacy scale [28], giving sub-scores for functional (e.g., “In reading instructions or leaflets from hospitals/pharmacies, you … (never/seldom/sometimes/often) found characters and words that you did not know”), communicative (e.g., “Since being diagnosed with diabetes, you have … (never/seldom/sometimes/often) collected information from various sources”) and critical health literacy (e.g., “Since being diagnosed with diabetes, you have … (never/seldom/sometimes/often) considered the credibility of the information”). Since health literacy in this study was used as an outcome measure of DSME, preference was given to a diabetes-specific measure of health literacy rather than a general measure, which is less sensitive to change as a result of an educational intervention.
(c) The perception of diabetes as a problem was measured using Problem Areas in Diabetes (PAID-5) [29] (e.g., “The next questions ask you which of the following diabetes issues are currently a problem for you: worrying about the future and the possibility of serious complications? (Not a problem/minor/moderate/somewhat serious/serious problem)”).
(d) Healthy coping was assessed by the Appraisal of Diabetes Scale (ADS) [30] (e.g., “How much uncertainty do you currently experience in your life as a result of being diabetic? (Not at all/slight/moderate/large/extremely large amount)”).
(e) Perceived health was measured using the “General Health Perception” subscale of the SF-36 [31] (e.g., “I seem to get sick a little easier than other people (definitely true—mostly true/do not know/mostly false/definitely false)”).
(f) Well-being was estimated via the WHO-5 Well-Being Index [32] (e.g., “I have felt cheerful and in good spirits (all of the time/most of the time/more than half of the time/less than half of the time/some of the time/at no time)”).

For the providers, implementation fidelity was assessed by means of a structured interview measuring the dimensions of implementation fidelity and potential influencing factors described by Carroll et al. [16]. The interview template, which had the format of a self-report questionnaire, was developed on the basis of a literature search, pilot tested for relevance with a group of French-speaking diabetes educators, and subsequently translated into the languages of the participating countries: English, German, Dutch, Hebrew and Mandarin Chinese. Respondents were asked to indicate the extent to which they had adhered to the content, duration, frequency and coverage of the intervention compared to the original protocol using a visual analogue scale. For each dimension, they were also asked to describe what the original program had been like, what it was like after the adaptation and why the program had been changed. The factors that might influence the fidelity of the implementation of an intervention were assessed by means of 5-scale Likert-type items (strongly disagree–strongly agree) grouped into: (a) participant-related factors (participants’ responsiveness, satisfaction and perception that the intervention met their needs) (11 items); (b) intervention complexity (10 items); (c) provider-related factors (quality of delivery) (12 items); (d) favorability of the context (4 items); and (e) availability and quality of facilitating strategies (training, intervention protocol, feedback and evaluation) (16 items). The interview template is available as supplementary material.

### 2.4. Statistical Analyses

Scale scores for the patients’ outcome scores and for the providers’ adherence to the program content, duration, frequency and coverage were obtained by calculating the mean score for the items of each scale, except for availability of facilitating strategies, for which a composite score was computed based on the availability and perceived quality of each strategy. A general adherence score was obtained by calculating the mean score for the four adherence dimensions. The scores for general adherence and each of the four dimensions were also dichotomized to differentiate between providers who had adapted the program and those who reported full adherence.

Internal consistencies of the scales measuring potential influencing factors were verified using Cronbach alpha coefficients, showing sufficient to good internal consistencies for the scales “participant-related factors” (α = 0.80) and “favorability of the context” (α = 0.72). For the scale “provider-related factors”, good internal consistency was obtained after the elimination of one item (α = 0.80). For “intervention complexity”, internal consistency was poor (α = 0.03), and hence no scale was constructed for this dimension. For “availability and quality of facilitating strategies”, no internal consistency coefficients could be calculated on account of the composite nature of the scores for this scale.

Exploratory analyses were performed using logistic regressions to test whether the potential influencing factors (participant- and provider-related factors, favorability of the context and facilitating strategies) were associated with the providers’ self-reported adherence to the program protocol, measured as a dichotomous variable for full adherence. Assumptions to perform logistic regressions (i.e., independence of the observations, exclusive and exhaustive categories of the dependent variable, a linear relationship between any continuous independent variable and the logit transformation of the dependent variable and no perfect or high multicollinearity between predictors) were checked and confirmed. As it is recommended to have at least 10 observations per independent variable [33], which was not the case in our sample, a first logistic regression was performed to predict the providers’ general adherence, followed by four other regressions to predict providers’ specific adherence to the content, duration, frequency and coverage of the intervention.

A second series of exploratory analyses was then performed using repeated measures MANOVA to evaluate the association between the providers’ adherence to (versus adaptation of) the original program and the program outcomes measured at patient level (diabetes health literacy, self-care behaviors, coping and perception of diabetes as a problem, general health and well-being). The assumptions to perform repeated measures MANOVA (i.e., a normal distribution for each dependent variable, a reasonable correlation between the dependent variable to avoid multicollinearity, homogeneity of variances when there is a between-group independent variable, homogeneity of the variance–covariance and sphericity of the within-group variances [34]) were verified. On that basis, it was decided to remove the items related to self-care medication as they did not meet the conditions. A first repeated measures MANOVA was conducted with the providers’ self-reported general adherence to the program protocol as an independent variable and controlling for social status and years of education. A second MANOVA considered the providers’ adherence to the content, duration, frequency and coverage separately.

## 3. Results

### 3.1. Descriptive Analyses

Of the 33 providers, 13 stated that they had fully adhered to the program protocol, while another 13 reported to have made at least one adaptation (10 reported changes in the content, 5 changed the duration, 9 adapted the frequency and 7 changed the coverage). Seven providers did not answer the questions on adherence (see Suppl. Table S2 for the patients’ characteristics depending on the provider reported adaptation of the program).

As only a few providers reported to have made adaptations, it was decided to create a dichotomized variable for adherence distinguishing between: (1) at least one adaptation, and (2) total adherence (no adaptation). Forty-nine patients had participated in the programs of the providers who reported at least one adaptation, and 80 in programs for which the providers reported total adherence. These two groups do not differ significantly from each other with regard to age (F(1,127) = 0.077, *p* = 0.784), gender (χ^2^(1) = 0.006, *p* = 0.939), years of schooling (F(1,127) = 1.225, *p* = 0.271) or social status (F(1,115) = 3.600, *p* = 0.060), but they do differ in terms of the representation of different nationalities. Most participants in the “adaptation” group are Israeli (65%), while participants in the “adherence” group are mainly English, American or Austrian.

### 3.2. Prediction of Provider Adherence to the Program

A first logistic regression analysis looking at the relation between the four potential influencing factors (participants’ and providers’ characteristics, favorability of the context and facilitating strategies) and the likelihood that providers would adapt the intervention did not show statistically significant results (χ^2^(7) = 7.63, *p* = 0.367) (Table 2). None of the predictor variables predicted the providers’ self-reported general adherence to the program (as opposed to adaptation). Logistic regressions using the four dimensions of provider adherence separately as predicted variables did not show a statistically significant association with the providers’ self-reported adherence to the content (χ^2^(4) = 5.51, *p* = 0.238), duration (χ^2^(4) = 5.99, *p* = 0.200), frequency (χ^2^(4) = 5.41, *p* = 0.248) or coverage (χ^2^(4) = 9.27, *p* = 0.055) of the program.

### 3.3. Impact of Provider Adherence on Program Outcomes

A first repeated measures MANOVA comparing the effects of diabetes self-management education programs with and without the providers’ general adherence on diabetes health literacy (DHL), self-care behaviors, diabetes coping, perception of diabetes as a problem, general health and well-being controlling for the participants’ social status and years of education showed a mean effect of the intervention for diabetes coping and for the perception of diabetes as a problem. Patient scores on these variables significantly improved after the intervention (F(2, 108) = 3.814, *p* ≤ 0.05). Changes for the other outcome variables were not significant. A multivariate interaction effect of time and adherence group was also observed, indicating that the effect over time was significantly different for the “adherence” and “adaptation” groups in terms of diabetes-specific health literacy (F(3, 98) = 4.651, *p* ≤ 0.01).

Univariate analyses indicated that the significant interaction effect was mainly due to critical diabetes health literacy (Table 3), whereby the improvement in critical diabetes health literacy was greater for participants of programs for which the provider had made adaptations than for those for which the provider had totally adhered to the original intervention (F(3, 89) = 13.397, *p* ≤ 0.001). Table 3 also shows several simple effects of adherence, indicating that before the intervention, the adherence group initially scored significantly higher for critical DHL (F(3, 89) = 4.068, *p* ≤ 0.05), exercise (F(3, 87) = 11.136, *p* ≤ 0.01), coping (F(2, 108) = 8.571, *p* ≤ 0.01), general health (F(2, 109) = 8.571, *p* ≤ 0.01) and well-being (F(2, 109) = 13.871, *p* ≤ 0.001), and lower for the perception of diabetes as a problem (F(2, 108) = 10.559, *p* ≤ 0.01).

A series of repeated measures MANOVAs with each of the four self-reported adherence dimensions (content, duration, frequency and coverage) as independent variables showed significant multivariate effects of the intervention for all of the intervention outcomes. Several multivariate interaction effects were found: (a) an interaction effect of time and adherence to the content on diabetes coping and perceiving diabetes as a problem (F(2, 81) = 5.214, *p* ≤ 0.01); (b) an effect of time and adherence to the duration on diabetes self-care behaviors (F(3, 63) = 3.300, *p* ≤ 0.001) and on general health and well-being (F(2, 77) = 6.113, *p* ≤ 0.01); (c) an effect of time and adherence to the frequency on diabetes coping and perceiving diabetes as a problem (F(2, 81) = 12.116, *p* ≤ 0.05); and (d) an effect of time and adherence to the coverage on diabetes health literacy (F(3, 58) = 3.080, *p* ≤ 0.05).

Subsequent univariate analyses (Table 4) showed that communicative diabetes health literacy decreased more when the provider reported adaptations to the program content (F(3, 58) = 4.372, *p* ≤ 0.05) or frequency (F(3, 58) = 7.775, *p* ≤ 0.01). On the other hand, an adaptation of the program content also led to a greater increase in critical diabetes health literacy (F(3, 58) = 4.900, *p* ≤ 0.05), as did an adaptation to the coverage (F(3, 58) = 8.275, *p* ≤ 0.01). In addition, an adaptation to the duration of the program was related to a greater improvement in dieting behavior (F(3, 63) = 10.089, *p* ≤ 0.005). Again, several simple effects are observed for adherence, indicating that before the intervention, the patients in the adherence groups scored significantly higher on outcomes such as communicative diabetes health literacy, coping, diet, general health and well-being.

## 4. Discussion

This study combined provider and patient level data of diabetes self-management programs implemented in eight different countries to document the fidelity with which the programs are implemented, and to evaluate the impact of implementation fidelity on program effectiveness. Implementation fidelity was defined as the providers’ self-reported adherence to the content, duration, frequency and coverage of the intervention, while program effectiveness was operationalized in terms of the participants’ improvement in diabetes health literacy, self-care behaviors, diabetes coping, perception of diabetes as a problem, general health and well-being. Factors related to the participants, the provider, the presence of facilitating strategies or the favorability of the context were considered as potential factors which determined the providers’ adherence to the program’s original protocol.

The results show that more than a third of the providers of diabetes self-management programs reported to have fully adhered to the intervention protocol. This proportion is surprisingly high, considering that the instrument that was used to measure implementation fidelity was developed to capture even small program adjustments. A possible explanation for this finding is that providers may overrate the adherence to the program protocol and may want to provide a favorable evaluation of the way they delivered the program [14,35,36]. The use of a self-report method to measure implementation fidelity, which is more prone to this type of distortion, may have exacerbated this tendency, despite the fact that the instructions explicitly referred to potential positive effects of adaptations as a strategy to overcome social desirability bias. Another explanation could be that the providers are not familiar with the intended content and scope of the intervention. Indeed, in the absence of consistent facilitating—or implementation—strategies [37] in the form of an intervention protocol, it is difficult for providers to assess their adherence to the intended intervention. While observational measures could have overcome this limitation, it was not possible to use observation in this study on account of its international scope and the number of programs involved. It would indeed have been very cumbersome to train enough observers to assess the implementation fidelity of this many programs in different countries. On the other hand, we also noted that providers who adapted the program and those who did not came from different countries. This may reflect cultural differences in the way instructions had been given to implement the program and/or the way program providers consider adherence or adaptation.

A second goal of this study was to identify the factors that contribute to the decision of providers of diabetes self-management to adapt their programs. Based on the model proposed by Carroll et al. [16], we assumed that adherence to a program would depend on factors related to the participants, to the provider, to the context and to the availability and quality of facilitating strategies such as a protocol, feedback or evaluation. Our findings do not confirm this assumption. This could be due to the small number of providers that were involved in the study. Ideally, logistic regression requires a sample size of at least 10 observations per predictor [33], which implies that a minimum of 40 providers would have been more suitable to test our model. On the other hand, there is hardly any empirical research available on the determinants of implementation fidelity, which means that the model we tested is a hypothetical one. As such, it is safe to conclude that the lack of support for the model in this study is an indication that the model itself needs to be refined, and that factors other than the ones we investigated may impact on program adherence.

Lastly, the comparison between programs with full adherence to the protocol and those that had made adaptations revealed that full adherence is not necessarily better, and that some adaptations can have a positive impact on some program outcomes. Specifically, adaptations of an intervention in terms of its content or coverage seem to be associated with a greater improvement in critical diabetes health literacy, while adaptations of the program duration give a greater improvement in the dieting behavior and general health of participants. Although the design of our study does not allow us to conclude whether these different effects can be attributed to the adaptation of the programs, or whether they are due to differences in the program content or group composition (e.g., in terms of nationality), these findings do suggest that adaptations may be positive. As such, they support the idea that it is relevant to distinguish between different kinds of program adaptations. In this regard, Stirman et al. [38] make a useful distinction between fidelity-consistent and fidelity-inconsistent adaptations. Fidelity-consistent modifications do not significantly alter the core components of the intervention, while fidelity-inconsistent ones reduce or remove components that are crucial to the nature of the intervention. It is likely that some of the reported adaptations, for instance, those with regard to the coverage (i.e., the number of participants required), are not associated with a decrease in program effectiveness, or that they can even increase effectiveness, as it is easier to reach all participants and engage them in the intervention in a smaller group. The latter is corroborated by findings of a recent meta-analysis which shows that benefits from chronic disease self-management are greater when fidelity requirements are unmet [39]. It is also the conclusion of qualitative studies suggesting that providers achieve better implementation when they are allowed to adjust the program [21]. Further qualitative research linking thematic categories of adaptation to effectiveness would indeed be interesting.

In accordance with the potential positive effects of program adaptations, some scholars have therefore proposed an extended version of the model of Carroll et al., which considers both fidelity and adaptation [23]. The idea is that adaptations, like adherence, can be assessed on several dimensions, and that both may be evaluated to identify the core ingredients that contribute to intervention effectiveness. Other authors [40] argue that fidelity and adaptation can be combined by involving the providers more actively in the program implementation and fidelity monitoring. This would imply that program developers consider providers as equal partners, provide them with the concepts and tools to identify the main components of the program and coach them in the process of adapting the intervention to local needs while maintaining the quality of the implementation [40]. Similar to the more familiar empowerment evaluation approach, such an “empowerment implementation” approach would have the additional benefit that providers can enhance their skills and capacities to implement programs in the future.

This study is not without its limitations. The small number of providers, the likely overestimation of program adherence and the different composition of provider groups in terms of nationalities do not allow us to draw far-reaching conclusions. Furthermore, programs were included from several countries, the results of which could not be analyzed separately due to the small numbers. On the other hand, the inclusion of programs implemented in different cultures and health systems adds to the ecological validity of our findings, since DSME is culturally sensitive [41]. It also adds variability to our sample, which, in order to test the effect of adaptation versus fidelity on program outcomes, is a positive element. Therefore, despite these limitations, we believe that our findings shed light on the importance of implementation as applied to DSME programs. It is the first study to assess implementation fidelity of DSME programs in different countries using a generic instrument. Furthermore, it provides an empirical view to the debate between proponents of a strict implementation fidelity approach and those who favor the adaptation of programs to the needs of participants and the local context. In this debate, our results tend to favor fidelity-consistent modifications.

## 5. Conclusions

While, thus far, studies in the field of implementation science have mainly focused on enhancing fidelity, our findings suggest that it is also worthwhile to consider adaptations of programs, provided that the conditions for effective adaptations are further clarified. The questionnaire used in our study, which was developed to assess the providers’ self-reported adherence to a program protocol, offers the opportunity to capture the nature of and the reasons for adaptations. A combined use of this tool with observational measures can highlight which type of health programs can benefit from adaptations and under which conditions.

## Figures and Tables

**Table 1:** Overview of the measures used in the study.

Questionnaire	Variables
Summary of Diabetes Self-Care Activities (SDSCA)	Diet
	Exercise
	Foot care
	Medication adherence
Diabetes Health Literacy scale (DHL)	Functional health literacy (diabetes-specific)
	Communicative health literacy
	Critical health literacy
Problem Areas in Diabetes (PAID-5)	Perception of diabetes as a problem
Appraisal of Diabetes Scale (ADS)	Coping with diabetes
SF-36 General Health Perception subscale	Perceived health
WHO-5 Well-Being Index	Well-being

**Table 2:** Logistic regressions predicting the likelihood of adaptation of the program by the provider based on participants’ engagement, providers’ attitude and knowledge, favorability of the context and presence of facilitating strategies.

	General Adherence				Adherence to the Content				Adherence to the Duration				Adherence to the Frequency				Adherence to the Coverage			
	% Correct	*R* ^2^	X²	*p*	% Correct	*R* ^2^	X²	*p*	% Correct	*R* ^2^	X²	*p*	% Correct	*R* ^2^	X²	*p*	% Correct	*R* ^2^	X²	*p*
Model	73%	0.332	7.63	0.367	62%	0.264	5.51	0.338	81%	0.299	5.99	0.200	71%	0.275	5.41	0.248	73%	0.482	9.27	0.055
			Exp(B)	*p*			Exp(B)	*p*			Exp(B)	*p*			Exp(B)	*p*			Exp(B)	*p*
Participants			0.070	0.054			0.439	0.545			0.392	0.485			0.434	0.176			1.51	0.883
Provider			0.795	0.860			0.041	0.110			0.240	0.496			7.42	0.548			2.77	0.570
Context			6.01	0.074			4.16	0.124			0.895	0.919			0.542	0.062			9.57	0.153
Facilitating strategies			1.23	0.136			1.00	0.988			1.31	0.079			1.31	0.531			1.81	0.069

**Table 3:** Repeated measures ANOVAs for program outcomes as a function of the providers’ general adherence to the program protocol.

Dependent Variable	Adherence (Mean and SD)		Adaptation (Mean and SD)		*F_time_*	*F_adherence_*	*F_interaction_*
	T_1_	T_2_	T_1_	T_2_			
Communicative DHL	2.91 (0.827)	2.83 (0.738)	2.99 (0.927)	2.75 (0.879)	0.008	0.018	1.063
Functional DHL	3.18 (0.684)	3.27 (0.557)	2.78 (0.828)	3.09 (0.695)	2.053	1.171	3.467
Critical DHL	3.09 (0.755)	3.06 (0.725)	2.59 (0.962)	2.89 (0.901)	6.375 *	4.068 *	13.397 ***
Diet	4.51 (1.592)	4.73 (1.306)	3.86 (1.679)	4.55 (1.35)	2.060	2.618	2.417
Exercise	2.97 (2.494)	3.54 (2.369)	1.64 (1.963)	2.68 (2.331)	2.464	11.136 **	2.336
Foot care	3.99 (1.647)	4.68 (1.285)	3.65 (1.848)	4.13 (1.653)	5.259 *	2.904	0.412
Problem	1.35 (1.092)	1.18 (1.068)	1.92 (1.231)	1.77 (1.311)	1.305	10.559 **	0.006
Coping	3.64 (0.624)	3.85 (0.675)	3.29 (0.698)	3.60 (0.789)	2.940	8.751 **	1.312
General health	3.24 (0.709)	3.41 (0.827)	2.79 (0.826)	3.10 (0.809)	1.113	10.032 **	3.931 *
Well-being	3.17 (1.112)	3.44 (3.234)	2.56 (1.271)	2.83 (1.183)	0.394	13.871 ***	0018

DHL, diabetes-specific health literacy. * *p* < 0.05, ** *p* < 0.01, *** *p* < 0.001.

**Table 4:** Repeated measures ANOVAs for program outcomes as a function of the providers’ adherence to the program protocol in terms of the content, duration, frequency and coverage.

	**Adherence** **(Mean and SD)**		**Adaptation** **(Mean and SD)**		***F_time_***	***F_adherence_***	***F_interaction_***
	**T_1_**	**T_2_**	**T_1_**	**T_2_**			
Adherence to the content							
Communicative DHL	3.03 (0.845)	2.87 (0.778)	2.88 (0.902)	2.66 (0.811)	7.408 **	5.063 *	4.372 *
Functional DHL	3.07 (0.761)	3.22 (0.567)	2.85 (0.837)	3.08 (0.765)	3.824	0.006	0.964
Critical DHL	3.00 (0.869)	3.03 (0.788)	2.54 (0.894)	2.91 (0.925)	13.855 ***	0.135	4.900 *
Diet	4.43 (1.724)	4.71 (1.405)	3.92 (1.555)	4.64 (1.609)	10.859 ***	0.636	1.694
Exercise	2.59 (2.600)	3.15 (2.519)	2.03 (1.683)	3.11 (2.194)	11.567 ***	0.536	0.931
Foot care	4.07 (1.676)	4.68 (1.426)	3.39 (1.859)	3.79 (1.468)	8.926 **	7.313 **	0.204
Problem	1.46 (1.194)	1.35 (1.160)	1.76 (1.099)	1.61 (2.324)	7.148 **	0.456	1.796
Coping	3.60 (0.680)	3.83 (0.713)	3.29 (0.654)	3.54 (0.746)	8.077 **	8.008 **	0.020
General health	3.10 (0.793)	3.34 (0.885)	2.97 (0.818)	3.19 (0.681)	1.81	0.409	1.051
Well-being	2.84 (1.375)	3.12 (1.254)	2.78 (1.193)	2.87 (1.107)	0.156	0.401	0.149
Adherence to the duration							
DHL communicative	2.99 (0.858)	2.84 (0.786)	2.74 (0.838)	2.65 (0.779)	7.408 **	5.709 *	0.744
DHL functional	3.05 (0.761)	3.21 (0.614)	2.93 (0.788)	3.18 (0.629)	3.824	0.141	1.854
DHL critical	2.92 (0.865)	3.02 (0.784)	2.85 (0.905)	2.92 (0.849)	13.855 ***	0.016	0.451
Diet	4.45 (1.620)	4.66 (1.416)	3.53 (1.576)	4.68 (1.539)	10.859 ***	1.963	10.089 **
Exercise	2.69 (2.471)	3.42 (2.439)	1.61 (1.847)	2.44 (2.011)	11.567 ***	3.365	0.064
Foot care	3.91 (1.794)	4.54 (1.421)	3.67 (1.456)	4.20 (1.572)	8.926 **	0.139	0.305
Problem	1.49 (1.164)	1.35 (1.206)	1.84 (1.197)	1.57 (1.152)	7.148 **	0.475	7.888
Coping	3.55 (0.678)	3.79 (0.735)	3.36 (0.641)	3.64 (0.692)	8.077 **	0.296	0.079
General health	3.13 (0.777)	3.35 (0.855)	2.81 (0.779)	3.11 (0.724)	1.81	0.419	0.146
Well-being	2.99 (1.291)	3.17 (1.242)	2.42 (1.307)	2.76 (1.101)	0.156	0.196	1.062
	**Adherence** **(Mean and SD)**		**Adherence** **(Mean and SD)**		***F_time_***	***F_adherence_***	***F_interaction_***
	**T_1_**	**T_2_**	**T_1_**	**T_2_**			
Adherence to the frequency							
DHL communicative	2.93 (0.860)	2.80 (0.774)	3.00 (0.862)	2.84 (0.831)	7.408 **	4.221 *	7.775 **
DHL functional	3.11 (0.701)	3.25 (0.550)	2.79 (0.911)	3.05 (0.777)	3.824	0.001	0.552
DHL critical	3.04 (0.763)	3.07 (0.739)	2,47 (1.049)	2.78 (0.937)	13.855 ***	0.900	0.302
Diet	4.31 (1.656)	4.67 (1.338)	4.13 (1.672)	4.60 (1.717)	10.859 ***	0.750	2.086
Exercise	2.56 (2.496)	3.26 (2.352)	2.22 (2.09)	3.02 (2.516)	11.567 ***	2.417	0.325
Foot care	3.87 (1.642)	4.60 (1.398)	3.87 (0.644)	4.11 (1.582)	8.926 **	2.681	1.962
Problem	1.55 (1.219)	1.28 (1.134)	1.62 (1.068)	1.77 (1.329)	7.148 **	0.036	2.996
Coping	3.55 (0.682)	3.79 (0.743)	3.37 (0.644)	3.65 (0.682)	8.077 **	1.545	1.247
General health	3.10 (0.792)	3.35 (0.831)	2.94 (0.759)	3.12 (0.835)	1.81	0.453	2.953
Well-being	2.85 (1.351)	3.13 (1.221)	2.76 (1.262)	2.88 (1.190)	0.156	0.632	1.247
Adherence to the coverage							
DHL communicative	2.91 (0.821)	2.75 (0.751)	3.34 (0.916)	2.99 (0.968)	7.408 **	3.691	2.059
DHL functional	3.21 (0.658)	3.25 (0.586)	2.57 (0.861)	2.98 (0.736)	3.824	3.390	2.391
DHL critical	2.98 (0.769)	2.97 (0.750)	2.50 (1.042)	2.72 (0.981)	13.855 ***	0.859	8.275 **
Diet	4.32 (1.549)	4.63 (1.396)	3.86 (1.806)	4.39 (1.732)	10.859 ***	1.235	0.587
Exercise	2.97 (2.223)	3.74 (2.245)	1.08 (1.893)	2.03 (2.324)	11.567 ***	16.108 ***	0.551
Foot care	3.79 (1.593)	4.34 (1.286)	3.61 (2.052)	4.31 (1.906)	8.926 **	0.147	0.293
Problem	1.44 (1.009)	0.99 (0.947)	2.16 (1.338)	2.23 (1.360)	7.148 **	0.1626	0.334
Coping	3.53 (0.608)	3.84 (0.675)	3.24 (0.759)	3.49 (0.887)	8.077 **	0.000	0.967
General health	3.20 (0.726)	3.43 (0.769)	2.61 (0.829)	2.91 (0.844)	1.81	12.833 ***	2.057
Well-being	3.07 (1.129)	3.28 (1.097)	2.28 (1.526)	2.55 (1.305)	0.156	7.510 **	0.071

DHL, diabetes-specific health literacy. * *p* < 0.05, ** *p* < 0.01, *** *p* < 0.001.

## Data Availability

The data presented in this study are available on request from the corresponding author. The data are not publicly available due to the different privacy and data storage regulations applied by the different organisations in the consortium.

## Supplementary Materials

The following are available online at https://www.mdpi.com/article/10.3390/ijerph18084095/s1: Table S1: Number of included programs and related providers and participants; Table S2: Participants’ characteristics depending on whether the provider reported at least one adaptation or full adherence or did not answer the questions about his/her adherence.
