# Supplementary material for: To Adapt or Not to Adapt: The Association between Implementation Fidelity and the Effectiveness of Diabetes Self-Management Education
Source: Int J Environ Res Public Health. 2021 Apr 13;18(8):4095. doi: 10.3390/ijerph18084095 (PMC8069177; doi:10.3390/ijerph18084095)
Supplement: Supplementary file 1 [file ijerph-18-04095-s001.pdf]

SUPPLEMENTARY MATERIALS

Table S1. Number of included programs and related providers and participants.

Country	N Programs in WP7	N Providers in WP7	N Patients in WP7
Austria	1	6	17
Belgium	2	2	3
Germany	3	3	19
Ireland	1	2	4
UK	3	3	50
Israel	2	11	44
Taiwan	2	4	10
USA	2	2	19
**Total**	**16**	**33**	**166**

Table S2. Participants' characteristics depending whether provider reports at least one adaptation, a total adherence or did not answer to any question about his/her adherence.

	At least one adaptation	Total adherence	Not apply/ No answer
Number of providers	13	13	7
Number of participants	49	80	37
Mean Age	60.6 (12.43)	61.2 (11.78)	61.34 (11.56)
Gender(F)	53%	54%	32%
Years of education	9.6 (4.37)	10.5 (4.74)	13.8 (3.64) [a]
Social status	5.8 (2.19)	5 (2)	5.6 (2.05) [b]
Main nationalities	Israeli (65%), German (28%)	English (29%), American (24%) and Austrian (19%)	English (73%) and German (14%)

[a] Years of education were significantly higher ($p \leq .001$) for the participants associated to a provider who did not answer to the questions about the adherence than for the two other groups

[b] The social status was significantly higher ($p \leq .001$) for the participants associated to a provider who did not answer to the questions about the adherence than for the participants associated to a provider who reported a total adherence
